# Seasonality of antenatal care attendance, maternal dietary intake, and fetal growth in the VHEMBE birth cohort, South Africa

**DOI:** 10.1371/journal.pone.0222888

**Published:** 2019-09-25

**Authors:** Carolyn A. Fahey, Jonathan Chevrier, Madelein Crause, Muvhulawa Obida, Riana Bornman, Brenda Eskenazi

**Affiliations:** 1 Center for Environmental Research and Children’s Health (CERCH), School of Public Health, University of California, Berkeley, California, United states of America; 2 Department of Epidemiology, Biostatistics and Occupational Health, McGill University Faculty of Medicine, Montréal, Québec, Canada; 3 School of Health Systems and Public Health, University of Pretoria Institute for Sustainable Malaria Control (UP ISMC), University of Pretoria, Pretoria, South Africa; Universidade Federal do Rio de Janeiro, BRAZIL

## Abstract

**Background:**

Seasonality of food availability, physical activity, and infections commonly occurs within rural communities in low and middle-income countries with distinct rainy seasons. To better understand the implications of these regularly occurring environmental stressors for maternal and child health, this study examined seasonal variation in nutrition and health care access of pregnant women and infants in rural South Africa.

**Methods:**

We analyzed data from the Venda Health Examination of Mothers, Babies and their Environment (VHEMBE) birth cohort study of 752 mother-infant pairs recruited at delivery from August 2012 to December 2013 in the Vhembe District of Limpopo Province, the northernmost region of South Africa. We used truncated Fourier series regression to assess seasonality of antenatal care (ANC) attendance, dietary intake, and birth size. We additionally regressed ANC attendance on daily rainfall values. Models included adjustment for sociodemographic characteristics.

**Results:**

Maternal ANC attendance, dietary composition, and infant birth size exhibited significant seasonal variation in both unadjusted and adjusted analyses. Adequate frequency of ANC attendance during pregnancy (≥ 4 visits) was highest among women delivering during the gardening season and lowest during the lean (rainy) season. High rainfall during the third trimester was also negatively associated with adequate ANC attendance (adjusted OR = 0.59, 95% CI: 0.40, 0.86). Carbohydrate intake declined during the harvest season and increased during the vegetable gardening and lean seasons, while fat intake followed the opposite trend. Infant birth weight, length, and head circumference z-scores peaked following the gardening season and were lowest after the harvest season. Maternal protein intake and ANC ≤ 12 weeks did not significantly vary by season or rainfall.

**Conclusions:**

Seasonal patterns were apparent in ANC utilization, dietary intake, and fetal growth in rural South Africa. Interventions to promote maternal and child health in similar settings should consider seasonal factors.

## Introduction

Seasonal food insecurity is a widespread yet overlooked phenomenon that particularly affects rural communities in low-and middle-income countries (LMIC) [1]. The annual pre-harvest “lean” season occurs when food stocks from the previous year’s harvest diminish and market prices rise, forcing households to reduce the quantity and nutritional quality of food consumed [2]. This period often coincides with the rainy season, a time of labor-intensive land preparation and planting along with a higher incidence of infectious and parasitic diseases. These conditions may impact the nutritional status of pregnant women, with potential consequences for fetal growth and health from infancy into adulthood [3]. Additionally, seasonal environmental stressors could constrain the ability of women to seek antenatal care during pregnancy.

A small but growing number of studies have demonstrated seasonality of birth size in LMICs, attributing these patterns to “seasonal energy stress” from concurrent food shortage, strenuous physical activity, and illness [4–9]. However, to our knowledge no studies have empirically examined the impact of seasonal conditions on antenatal care (ANC) attendance during pregnancy. Global efforts to improve maternal and child health from the Millennium Development Goals (MDGs) to the Sustainable Development Goals have sought to expand access to ANC for routine monitoring and prevention of pregnancy complications, including nutritional supplementation and counseling. Still, only 64% of women globally and 48% of women in Africa from 2007–2014 receive the MDG targeted minimum of four ANC visits [10]. Seasonal barriers to ANC attendance may include poor road conditions during heavy rainfall, availability of resources to pay for transportation, low energy, and opportunity cost of time away from subsistence farming or other work [11]. As ANC presents a key opportunity to detect and intervene on pregnancy complications, including seasonal stressors, it is critical to understand whether ANC attendance is itself limited by seasonality. To address this gap, this study examined seasonal trends in ANC attendance along with maternal dietary intake and infant birth size in rural South Africa.

## Materials and methods

### Study setting and population

We analyzed data from the Venda Health Examination of Mothers, Babies and their Environment (VHEMBE) study conducted in the Vhembe District of Limpopo Province, the northernmost region of South Africa. Many households in this area engage in subsistence crop production as a source of food, growing primarily maize, fruits, and vegetables in backyards or on small plots of land [12]. The climate is subtropical with rainfall concentrated during a four-month period (November–February) [13]. The annual farming cycle revolves around rainfall: planting of staple crops occurs at the onset of the rainy season, followed by harvesting (March–June) after rainfall ends and vegetable gardening (July–October) until rainfall commences again [14,15]. As food stocks from the harvest become depleted over the year and market prices rise, a period of widespread food insecurity known as the “lean” season (November–February) coincides with rainfall, labor-intensive land preparation and planting, and an increase in infectious and parasitic diseases [1,15].

The VHEMBE study is a prospective birth cohort that aims to evaluate the determinants and impacts of environmental exposures on child growth, health, and development. This analysis used data collected at the time of delivery, including abstracted medical records that were recorded throughout the pregnancy. Participants were recruited among women who presented for delivery at Tshilidzini hospital, in the town of Thohoyandou, between August 2012 and December 2013. All women delivering or still in the hospital on a weekday were screened (n = 1,649). Women were eligible for inclusion if they were at least 18 years old, had contractions at least five minutes apart, spoke primarily TshiVenda (the most commonly spoken language in the area), lived within 20 km of the hospital and intended to remain in the area for at least two years, had not been diagnosed with malaria during the pregnancy and gave birth to a viable singleton. Of 920 eligible women, 152 declined to participate and 16 were lost to follow-up before full enrolment data were collected, yielding a study sample of 752 mother-infant pairs. For this analysis, sample sizes ranged from 605 to 751 due to missing outcomes data including first ANC visit date (n = 147), total number of ANC visits (n = 140), birth length (n = 6), head circumference (n = 6), birth weight (n = 1), and dietary intake (n = 1).

Following recruitment at the time of delivery, trained bilingual Venda interviewers administered a structured questionnaire to collect sociodemographic characteristics and abstracted medical records. Participants were also visited at home one week after delivery, where Global Positioning System (GPS) coordinates were captured using a Garmin Etrex30 device outside the front door of the building where the mother slept. Coordinates were taken in duplicate (midway through the visit and at the end) and averaged to assign the final coordinates of each home.

The University of California, Berkeley, the University of Pretoria, the Limpopo Department of Health and Social Development, Tshilidzini Hospital and McGill University granted ethics approval for the VHEMBE study. The Committee for Protection of Human Subjects at the University of California, Berkeley further approved the use of these data for the present study.

### Outcome variables

#### Antenatal care attendance

Antenatal care (ANC) attendance records were collected at delivery from the mother’s ANC card, a form of medical record which women carry with them to all visits to any ANC clinic during pregnancy. We characterized ANC attendance by adequate frequency (≥4 visits) and early initiation (≤12 weeks), according to WHO recommendations at the time of the study [16]. Records of ANC attendance were missing for 140 (19%) of mothers. Field staff reported that mothers often forgot or did not have time to retrieve their ANC card from home and bring it to delivery. Although it is possible that some of these mothers never attended ANC, this proportion is expected to be small (nationally, 97% of mothers attend at least one ANC visit [17]). However, to limit potential bias from missing ANC records, we applied inverse probability of censoring weighting to ANC analyses (see Statistical Analysis) [18].

#### Dietary intake

During the interview at the time of delivery, mothers completed a detailed quantitative food frequency questionnaire designed and validated in a population residing in the study area, which asked about food consumption in the previous month [19]. Total and per macronutrient energy intake in kilojoules (kJ) was estimated by a South African expert nutritionist using FoodFinder 3 software (South Africa Medical Research Council/WAMTechnology CC). For this analysis, we calculated carbohydrate, fat, and protein intakes as percentages of total energy intake.

#### Infant birth size

Child birthweight was assessed by hospital nurses immediately after delivery—with a Tanita BD-815U neonatal scale (Arlington Heights, IL, USA) provided by the study which measured grams to two decimal places—and was retrospectively abstracted from hospital records. Study nurses measured birth length and head circumference within the first 24 to 48 hours of birth. Birth length was measured using a Seca 417 portable infantometer (Chino, CA, USA), with the infant laid on his or her back, legs aligned and fully extended, and toes pointing directly upward. Head circumference was assessed to the nearest 0.1 cm using a tape measure positioned just above the eyebrows, above the ears, and around the largest part of the back of the head. Measurements were taken in triplicate and averaged. For each size outcome, growth z-scores for gestational age and sex were constructed according to WHO Child Growth Standards [20]. Gestational age was ascertained by last menstrual period (LMP) reported at delivery, with unlikely values (above the 1^st^ or below the 99^th^ birthweight-for-age percentiles) replaced by gestational age indicated in medical records for 10% of participants. Birth size measurements were complete for all but one missing birth weight and six missing length and head circumference measurements.

### Statistical analysis

We used logistic regression to model ANC outcomes (adequate attendance and early initiation) and linear regression for dietary intake (carbohydrate, fat, and protein) and birth size outcomes (birth weight, birth length, and head circumference z-scores) in relation to season. Seasonal trends in ANC attendance, dietary intake, and birth size were examined using truncated Fourier series terms [21], a method previously used and recommended by Rayco Solon et al. [5] and Fulford et al. [22] who have conducted extensive work on seasonality of birth size. This method avoids the pitfalls of using categorical seasons or month-of-year predictors, including arbitrary cutoffs and overparameterization. Delivery dates were transformed into cyclical data using sine and cosine functions parameterized by Fourier coefficients, allowing for modeling of continuous trends in seasonality. Borrowing from previous notation [5,22], we defined seasonal terms as follows:

$$\sum_{r=1}^{p}\beta_{r}\;\text{sin}\left(r\theta_{i}\right)+\gamma_{r}\;\text{cos}\left(r\theta_{i}\right),$$

where *p* is the number of Fourier term pairs assessed and *θ*_*i*_ is the point in the annual cycle when the *i*^*th*^ infant is born, calculated in radians (starting with the harvest, we set March 1 ~ 0; February 28 ~ 2π). Seasonality is modeled by adding the first *p* pairs of Fourier terms to the regression model, parameterized by *β*_*r*_ and *γ*_*r*_. Separate models for each ANC attendance, dietary intake, and infant birth size outcome were fitted with an increasing number of Fourier pairs. Seasonality was assessed using likelihood ratio (LR) tests or *F* tests for multiply imputed data to compare models and select the model with the best fit for each outcome. For each outcome, we selected the model that satisfied α = 0.10 for both comparison to the null model (no Fourier terms) as well as to the nested model with fewer Fourier terms.

As a secondary analysis, we explored the role of rainfall in ANC attendance. Rainfall during each pregnancy was constructed with the Climate Hazards Group InfraRed Precipitation with Station (CHIRPS) daily data at 0.05° resolution [23]. CHIRPS is a publicly available quasi-global rainfall dataset that uses satellite imagery along with in-situ station data to create gridded rainfall data spanning 50°S to 50°N from 1981 to near-present. We averaged precipitation values within a 0.25° radius (~30 km) of a given woman’s home for each day of her pregnancy, summed across each trimester, and divided by the number days in each trimester (S1 Fig). Adequate ANC attendance was regressed on the average daily amount of rainfall during each trimester. Early ANC initiation was regressed on rainfall during the first trimester only. Average daily rainfall per trimester was modeled both as a continuous variable and in separate models as a categorical indicator for above or below the sample average.

All regression models were adjusted for covariates identified *a priori* from the literature [5,6,24,25]. Models of ANC attendance included maternal parity, HIV status, education, marital status, and pregnancy desire; father’s supportiveness of the pregnancy; household income and distance from the home to the nearest main road (calculated as the Euclidian distance to a primary or secondary road obtained from OpenStreetMap [26]); and duration of pregnancy. Models of dietary intake included maternal parity, HIV status, height, education, marital status, household income, and duration of pregnancy. Models of birth size z-score included maternal parity, HIV status, height, education, marital status, and household income. Covariates were coded as shown in Table 1 except for income, which was log transformed.

**Table 1:** Characteristics of mother-infant pairs by season of birth, VHEMBE study, South Africa, 2012–2013.

			Season of birth			
	Total	Harvest (Mar-Jun)	Gardening (Jul-Oct)	Lean/Rainy (Nov-Feb)	p-value	Test
	N = 752	N = 339	N = 174	N = 239		
Sex of child					0.71	Pearson’s χ^2^
Boy	388 (51.6)	178 (52.5)	85 (48.9)	125 (52.3)		
Girl	364 (48.4)	161 (47.5)	89 (51.1)	114 (47.7)		
Gestational age (weeks)	39.27 (2.3)	39.12 (2.3)	39.57 (2.1)	39.26 (2.5)	0.11	ANOVA
Maternal age					0.95	Pearson’s χ^2^
18–21 years	233 (31.0)	105 (31.0)	52 (29.9)	76 (31.8)		
22–29 years	316 (42.0)	140 (41.3)	73 (42.0)	103 (43.1)		
≥ 30 years	203 (27.0)	94 (27.7)	49 (28.2)	60 (25.1)		
Parity					0.15	Pearson’s χ^2^
First child	326 (43.4)	145 (42.8)	81 (46.6)	100 (41.8)		
Second child	201 (26.7)	94 (27.7)	34 (19.5)	73 (30.5)		
≥ Third child	225 (29.9)	100 (29.5)	59 (33.9)	66 (27.6)		
Maternal HIV status					0.08	Pearson’s χ^2^
HIV Negative	645 (86.1)	288 (85.0)	157 (91.3)	200 (84.0)		
HIV Positive	104 (13.9)	51 (15.0)	15 (8.7)	38 (16.0)		
Maternal height (cm)	158.2 (6.8)	157.7 (5.9)	157.7 (7.3)	159.2 (7.4)	0.02	ANOVA
Maternal education					0.19	Pearson’s χ^2^
< Secondary	412 (54.9)	187 (55.2)	92 (52.9)	133 (55.9)		
Secondary	229 (30.5)	94 (27.7)	55 (31.6)	80 (33.6)		
Further studies	110 (14.6)	58 (17.1)	27 (15.5)	25 (10.5)		
Marital status					0.58	Pearson’s χ^2^
Not married	392 (52.2)	177 (52.2)	96 (55.2)	119 (50.0)		
Married or living as married	359 (47.8)	162 (47.8)	78 (44.8)	119 (50.0)		
Mother’s pregnancy desire					0.56	Pearson’s χ^2^
Wanted a baby now	302 (40.2)	140 (41.3)	73 (42.0)	89 (37.4)		
Untimed or unintended	449 (59.8)	199 (58.7)	101 (58.0)	149 (62.6)		
Father’s support during pregnancy					0.34	Pearson’s χ^2^
Very supportive	560 (74.6)	250 (73.7)	137 (78.7)	173 (72.7)		
Somewhat supportive or less	191 (25.4)	89 (26.3)	37 (21.3)	65 (27.3)		
Household monthly income (Rand)	2000 (1280–3500)	2070 (1500–3860)	2000 (1280–3700)	1825 (1000–3000)	0.02	Kruskal-Wallis
Distance from home to main road (km)	1.44 (0.7–2.9)	1.35 (0.6–2.6)	1.56 (0.5–3.4)	1.51 (0.8–2.9)	0.22	Kruskal-Wallis

Data are presented as mean (SD) or median (IQR) for continuous measures, and n (%) for categorical measures. Variables with missing data: distance to a main road (n = 30), maternal height (n = 12), household income (n = 4), maternal HIV status (n = 3), and maternal education, marital status, pregnancy desire and father’s supportiveness (n = 1 for each).

To limit potential bias from missing ANC data, we used inverse probability of censoring weighting (IPCW) to adjust for the propensity of missing ANC records [18]. We generated weights from a logistic model of missing versus observed ANC records regressed on the characteristics shown in S1 Table. This model incorporated multiple imputation by chained equations (MICE) with 20 iterations for predictors with missing data [27, 28], using the same variables shown in S1 Table. We also used these multiple imputation estimates in our adjusted outcomes regression models to include participants with missing covariates that would otherwise drop out of the model (n = 21 for ANC models; n = 15 for dietary intake models and birth weight; n = 17 for birth length and head circumference). Results were combined according to Rubin’s rules using the “mi estimate” command in Stata [27].

All statistical analyses were conducted using Stata 14.2 (College Station, TX, USA). Rainfall variables were constructed using MATLAB Release 2018b (Natick, MA, USA) and road distance was calculated using R Version 3.5.1 (Vienna, Austria).

## Results

### Mother and infant characteristics

Among 752 singleton births, 52% of infants were male and the mean gestational age at birth was 39.3 weeks (SD: 2.3). The mean age of mothers at delivery was 26.4 years (SD: 6.3), 43% were primiparous, 55% had not completed secondary school, and 14% were HIV-positive. Maternal characteristics varied by categorical season of delivery, including lower reported typical monthly household income and taller stature among women giving birth during the lean, rainy season (Table 1).

Overall, 16% of women initiated ANC in the first 12 weeks of pregnancy and 73% attended at least four total ANC visits during pregnancy (Table 2). The average dietary composition in the month before giving birth included 61% of energy from carbohydrates (SD: 10.0), 24% from fat (SD: 8.2), and 13% from protein (SD: 2.9). The mean infant size measurements included birth weight of 3125 g (SD: 452.1), length of 48.9 cm (SD: 2.23), and head circumference of 34.4 cm (SD: 1.49).

**Table 2:** Summary of outcomes for antenatal care, maternal dietary intake, and infant birth size, VHEMBE study, South Africa, 2012–2013.

Outcome	N	Summary
Antenatal care attendance		
≥ 4 total visits	612	454 (73.3%)^a^
First visit ≤ 12 weeks	605	98 (16.2%)^a^
Maternal diet (% of energy)		
Carbohydrate	751	61 (10.0)
Fat	751	24 (8.2)
Protein	751	13 (2.9)
Infant birth size		
Birth weight		
grams	751	3125 (452.1)
z-score	751	-0.51 (1.19)
Birth length		
cm	746	48.9 (2.23)
z-score	746	-0.57 (1.13)
Head circumference		
cm	746	34.4 (1.49)
z-score	746	-0.0 (1.18)

Data are presented as mean (SD) for continuous measures, and n (%) for categorical measures.

^a^. Percentages adjusted using inverse probability of censoring weighting to account for missing data.

### Seasonality of antenatal care, diet, and birth size

**Fig 1:**
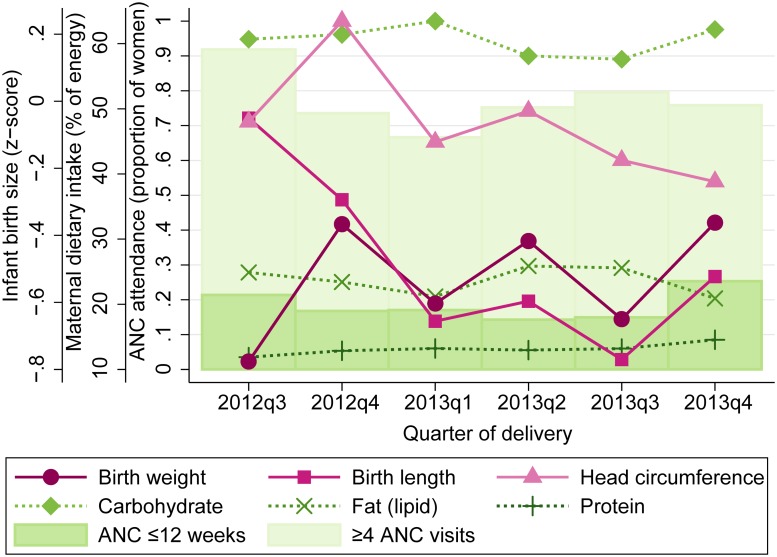
Maternal dietary intake, antenatal care attendance, and infant birth size across the study period, VHEMBE study, South Africa, 2012–2013. Data are means by quarter-year of delivery for maternal carbohydrate, fat, and protein intake as a percentage of total energy in the past month; proportion attended ANC ≤12 weeks gestation and ≥4 total ANC visits during pregnancy; and birth weight, length, and head circumference z-scores standardized by gestational age and sex.

Descriptive trends in maternal ANC attendance, dietary intake, and infant birth size are presented in Fig 1 for the duration of study enrollment period, from the first delivery in August 2012 to the final delivery in December 2013. In adjusted analyses of seasonality, Fourier-transformed date of delivery was associated with attendance at ≥4 ANC visits during pregnancy, carbohydrate and fat intake in the past month, and infant birth weight, length, and head circumference (Table 3). The model including the first and second order pair of Fourier terms (bimodal shape) provided the best fit for outcomes including ≥4 ANC visits (p = 0.05 vs. null; p = 0.09 vs. 1^st^ order), maternal carbohydrate (p<0.01 vs. null; p = 0.05 vs. 1^st^ order) and fat (p<0.01 vs. null; p = 0.04 vs. 1^st^ order) intake, and birth weight (p = 0.06 vs. null; p = 0.01 vs. 1^st^ order), length (p<0.01 vs. null; p<0.01 vs. 1^st^ order), and head circumference (p = 0.04 vs. null; p = 0.02 vs. 1^st^ order). Early ANC initiation and protein intake did not vary by date of delivery; in other words, no combination of Fourier terms provided a better fit than the null model without Fourier terms. The seasonal associations for each outcome from adjusted models were somewhat stronger than from unadjusted models (S2 Table).

**Table 3:** Comparison of truncated Fourier series models for seasonality of antenatal care attendance, maternal dietary intake, and infant birth size, VHEMBE study, South Africa, 2012–2013.

		1st order (unimodal)		1st-2nd order (bimodal)				1st-3rd order (trimodal)			
		vs. Null:		vs. Null:		vs.1st order:		vs. Null:		vs. 1st-2nd order:	
Outcome	N^d^	*F* (df)	p-value	*F* (df)	p-value	*F* (df)	p-value	*F* (df)	p-value	*F* (df)	p-value
Antenatal care attendance^a^											
≥ 4 total visits	612	2.63 (2)	0.07	2.40 (4)	0.05	2.47 (2)	0.09	2.10 (6)	0.05	1.26 (2)	0.29
First visit ≤ 12 weeks	605	1.00 (2)	0.37	0.97 (4)	0.42	0.87 (2)	0.42	0.74 (6)	0.62	0.47 (2)	0.62
Maternal diet (% of energy)^b^											
Carbohydrate	751	30.1 (2)	<0.01	16.6 (4)	<0.01	2.97 (2)	0.05	11.9 (6)	<0.01	2.25 (2)	0.11
Fat	751	32.1 (2)	<0.01	17.8 (4)	<0.01	3.24 (2)	0.04	12.7 (6)	<0.01	2.32 (2)	0.10
Protein	751	1.32 (2)	0.27	0.91 (4)	0.46	0.50 (2)	0.61	1.44 (6)	0.20	2.49 (2)	0.08
Infant birth size (z-score)^c^											
Birth weight	751	0.20 (2)	0.82	2.29 (4)	0.06	4.38 (2)	0.01	1.68 (6)	0.12	0.47 (2)	0.63
Birth length	746	4.33 (2)	0.01	5.01 (4)	<0.01	5.64 (2)	<0.01	3.65 (6)	<0.01	0.92 (2)	0.40
Head circumference	746	1.24 (2)	0.29	2.57 (4)	0.04	3.88 (2)	0.02	1.92 (6)	0.07	0.65 (2)	0.52

Data are likelihood ratio or *F* test statistics comparing generalized linear models regressed on Fourier terms for date of birth. Four models were compared for each outcome: (1) intercept plus covariates only (Null model); (2) including the first order Fourier pair (unimodal); (3) including the first and second order Fourier pairs (bimodal); and (4) including the first through third order Fourier pairs (trimodal). *F* tests were used to jointly test Fourier coefficients for each nested model.

^a^. Models adjusted for maternal parity, HIV status, education, marital status, and pregnancy desire; father’s supportiveness of the pregnancy; household income and distance to a main road; and duration of pregnancy. Inverse probability of censoring weights applied to adjust for missing data.

^b^. Outcomes are dietary intake type as a percentage of total energy (kJ) in the month before delivery. Models adjusted for maternal parity, HIV status, height, education, and marital status; father’s supportiveness of the pregnancy; household income; and duration of pregnancy.

^c^. Outcomes are gestational age- and sex-adjusted z-scores. Models adjusted for maternal parity, HIV status, height, education, and marital status; and household income.

^d^. Number of participants included in each model, as determined by observed outcome data. Multiple imputation was used to impute missing covariates.

**Fig 2:**
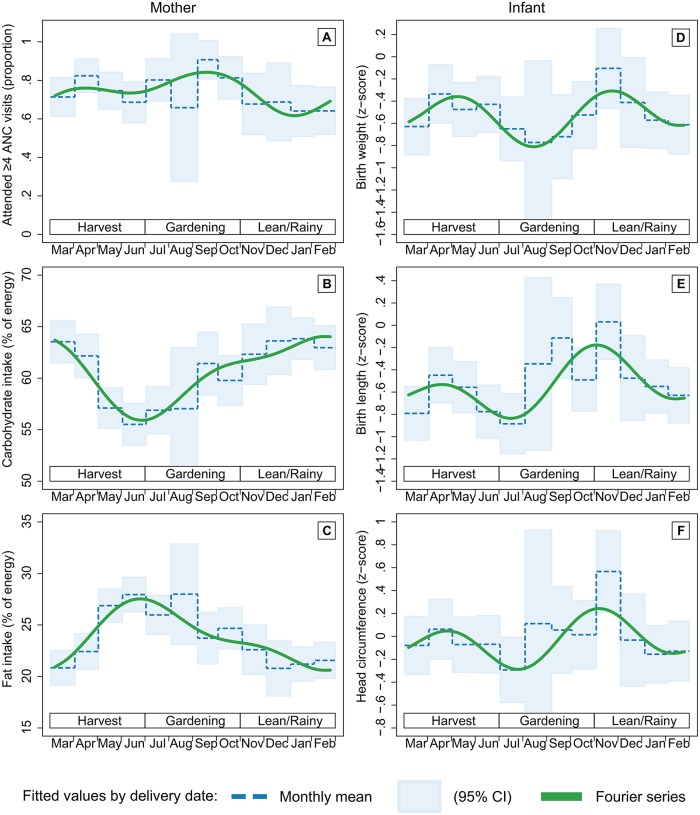
Fitted values of antenatal care (ANC) attendance, maternal dietary intake, and infant birth size by date of delivery, VHEMBE study, South Africa, 2012–2013. Fourier series models were fitted with first and second order terms for (A) attendance at 4 or more ANC visits during pregnancy; (B) maternal carbohydrate and (C) fat intake as a percent of total energy in the month before delivery; and (D) infant birth weight, (E) length, and (F) head circumference z-scores standardized by gestational age and sex.

Based on the peaks and troughs in the Fourier series fit (Fig 2), attendance at ≥4 ANC visits was highest among mothers who gave birth during the vegetable gardening season (peak: 84% in September), then declined by 20 percentage points over the next 4 months to a low during the lean, rainy season (trough: 64% in January). Carbohydrate intake as a percentage of total energy was highest during the lean season (peak: 64% in January) and lowest at the end of the harvest season (trough: 56% in June); fat followed the opposite trend, with the lowest intake during the lean season (trough: 21% in January) and the highest at end of the harvest season (peak: 28% in June). Birth size z-scores peaked in November at the onset of the lean season (birth weight: -0.31; length: -0.18; head circumference: 0.24), declined during the lean season until another smaller peak during the harvest season, then reached a trough at the onset of the gardening season (birth weight: -0.81 in August; length: -0.84 in July; head circumference: -0.29 in July).

### Rainfall and antenatal care

Pregnant women exposed to above average daily rainfall during the third trimester (after week 26) had lower odds of attending ≥4 ANC visits during pregnancy than those exposed to lower rainfall (adjusted OR (OR_a_) = 0.59, 95% CI: 0.40 to 0.86; Table 4). This association was also evident when modeling the exposure as a continuous variable for increasing mean rainfall (mm) per day (OR_a_ = 0.89, 95% CI: 0.82 to 0.96; S3 Table). There was no significant association between first trimester rainfall and early ANC initiation (≤12 weeks) for categorical (OR_a_ = 0.83, 95% CI: 0.54 to 1.30) or continuous (OR_a_ = 0.92, 95% CI: 0.77 to 1.11) rainfall characterizations.

**Table 4:** Relationship between high rainfall during each trimester of pregnancy and antenatal care (ANC) attendance, VHEMBE study, South Africa, 2012–2013.

		1st trimester rainfall				2nd trimester rainfall				3rd trimester rainfall			
		Unadjusted		Adjusted		Unadjusted		Adjusted		Unadjusted		Adjusted	
ANC attendance	N	OR	(95% CI)	OR	(95% CI)	OR	(95% CI)	OR	(95% CI)	OR	(95% CI)	OR	(95% CI)
≥ 4 total visits	612	1.30	(0.90 to 1.88)	1.27	(0.87 to 1.86)	1.15	(0.79 to 1.67)	1.14	(0.78 to 1.68)	0.64	(0.44 to 0.93)	0.59	(0.40 to 0.86)
First visit ≤ 12 weeks	605	0.83	(0.54 to 1.30)	0.79	(0.50 to 1.23)	–		–		–		–	

Data are odds ratios (OR) and 95% confidence intervals (CI) from logistic regression models of binary antenatal care (ANC) attendance outcomes regressed on average daily rainfall (binary: above vs. below sample average) for each trimester. Inverse probability of censoring weights were applied to all models to account for missing outcomes data. Adjusted models include maternal parity, HIV status, education, marital status, and pregnancy desire; father’s supportiveness of the pregnancy; and household income and distance to a main road.

## Discussion

In a rural South African cohort of mother-infant pairs recruited at delivery, we identified significant seasonal variation in antenatal care (ANC) attendance, maternal dietary intake, and infant birth size. Truncated Fourier series models for seasonality showed that adequate ANC attendance (≥4 visits) was lowest among mothers delivering during the “lean” season when food insecurity, agricultural labor, and rainfall typically occur. This trend corresponded with another finding that higher average daily rainfall during the third trimester correlated with a decline in adequate ANC attendance. We also identified seasonal trends in the composition of maternal diets, including a trade-off of carbohydrates for higher fat intake during the harvest season, when food prices are generally lower. Additionally, we found that birth weight, length, and head circumference decreased during the lean season and increased during the post-harvest vegetable gardening season. This study adds to previous research on birth size and seasonality, and to our knowledge is the first to model the influences of seasonality or rainfall on ANC attendance in a low- to middle-income country (LMIC) setting.

A review of birth size and seasonality in LMICs concluded that such patterns are likely due to seasonal variation in food security, agricultural labor, and infectious disease [9]. For example, Rayco-Solon et al. [5] used Fourier series to show seasonality of small-for-gestational-age (<10^th^ percentile) among 1,916 live births over 26 years in rural Gambia, with the highest incidence at the end of the annual lean season and rates varying inversely with maternal weight changes across the year. In a prospective cohort including 633 full-term births rural India, Rao et al. [6] found that birth weight and length decreased during the rainy season and increased after the harvest. They also associated increasing birthweight with higher maternal intake, lower physical activity, and longer duration of exposure to the harvest season (when dietary intake was highest). Our findings similarly show that birth size falls during the rainy (lean) season—the time of highest food insecurity, agricultural labor, and infection—and peaks during the harvest and again after the gardening season. As most fetal growth occurs later in pregnancy—and inadequate second or third trimester weight gain has been associated with reduced fetal growth [29–31]—these findings bolster the hypothesis that annual agricultural and climatologic cycles influence birth size in LMIC.

Recent work suggests that seasonality of pregnancy outcomes may be partly attributable to seasonal variation in the characteristics of pregnant women, confounding potential causation by seasonal environmental conditions. A study among U.S. women demonstrated that differences in socioeconomic status by pregnancy timing contributed to seasonal variation in the gestational age distribution [32]. However, another U.S. study showed persistent seasonality of birthweight using a within-mother analysis of 647,050 groups of siblings to control for maternal characteristics [33]. While noting that the drivers of birth outcome seasonality likely differ in high income countries with lower reliance on agriculture, in this LMIC setting we did identify some differences in maternal characteristics by birth season such as height and household income. By controlling for these factors, our analyses strengthen the evidence for environmental drivers of pregnancy outcomes in LMICs.

Promising interventions to mitigate seasonal declines in fetal growth are suggested by randomized controlled trials in rural Gambia [4] and Burkina Faso [8], which both showed that nutritional supplementation during the rainy (lean) season is particularly effective at increasing birth size. Routine ANC visits could provide a platform for scaling up nutritional supplementation during the lean season, while also preventing and treating infectious diseases such as malaria and HIV that negatively influence fetal growth [34]. However, our results indicate a seasonal decline in adequate ANC attendance for births during the rainy (lean) season. We found similar trends when investigating actual rainfall during the third trimester as the exposure, suggesting that rainfall and accompanying poor road conditions may impede ANC attendance. We did not find any association with early ANC initiation; the rarity of this outcome (16%) is consistent with prior studies and suggests that previously identified factors such as socioeconomic barriers, knowledge and attitudes, and previous or anticipated adverse experiences with ANC may have a larger role in delaying ANC than seasonal conditions [35–37]. Women in this study tended to initiate ANC late in pregnancy, with the average first visit during week 20 and the third and fourth visits during the third trimester. This late attendance may contribute to lower completion of four ANC visits when the third trimester occurs during the rainy season.

Our finding of an association between rainfall and adequate ANC attendance is supported by qualitative studies in similar settings, such as the following quote from a study in rural Indonesia:

*“It is really hard when it is raining*. *We are afraid we will fall over because the road is so slippery and we are pregnant*. *The health centre is far and you can see that the road is poor*.*”*

[38]

Women experiencing hunger during the lean season may have little energy to attend ANC, particularly for women who must walk far in wet conditions to reach the clinic or a road where they can take public transportation. Travel costs or other fees may also be particularly burdensome during the lean season when household resources are limited. Other seasonal barriers may include the opportunity cost of time away from household resource generation, particularly during critical times in the subsistence farming cycle when missed labor could imperil the household food supply [11,39,40]. Together with qualitative insights, our findings suggest that efforts to increase ANC should focus on the rainy (lean) season. For example, transportation vouchers or conditional cash transfers—which have been shown to increase ANC attendance [41]—or mobile clinics could be offered during the rainy season. Additionally, ANC providers could target nutritional supplementation during this time to promote fetal growth. Findings from this study highlight the need for awareness and interventions to address seasonality of ANC attendance, maternal nutrition, and fetal growth.

### Strengths and limitations

This study was not designed to identify specific causal pathways that may operate between seasonal factors and ANC, nutrition, or birth size. As mothers were enrolled at the time of delivery, some variables of interest including maternal health and activity levels were not measured prospectively during the pregnancy. While a handful of other studies have documented changes in maternal caloric intake, physical activity, and illness that may explain seasonality in birth size, further research is needed to disentangle the factors contributing to seasonality of ANC.

Our results are also limited by a relatively short observation period of 17 months, which may not represent long-term seasonal patterns in the community. Additionally, given the adolescent fertility rate (ages 10–19) of 65 per 1,000 births in Limpopo Province [42], this study excluded a small but vulnerable portion of the population and therefore may somewhat underestimate adverse seasonal trends. While the specific monthly trends identified in this study may not generalize to other regions due to differences in climate and population, many LMIC settings similarly experience an annual lean season with the potential to adversely influence maternal and child health and nutrition.

Misclassification of gestational age may have occurred from calculation based on mothers’ recollection of last menstrual period at the time of delivery. However, we attempted to reduce measurement error by cross-referencing with medical records of gestational age based on earlier reported LMP or fundal height. Finally, missing ANC data for 19% of participants likely reduced the precision of our estimates and introduced potential bias due to differing characteristics of participants missing ANC records. We addressed this issue using inverse probability of censoring weighting, however it is possible that this approach did not fully account for systematic differences.

## Conclusions

Despite these important limitations, our analysis strengthens the evidence for seasonality of birth size and maternal nutrition while contributing to a dearth of knowledge about seasonality of ANC attendance, especially in LMIC settings. These results have implications for the potential effects of climatic events—including increasing frequency and magnitude of extreme events such as flooding and drought [43]—on health care access, food availability, and maternal and child health. Further research should prospectively investigate maternal dietary intake and food insecurity, physical activity, and illness in relation to ANC attendance and birth outcomes across multiple years to better understand causal mechanisms and opportunities for intervention.

## Supporting information

S1 Fig Construction of rainfall estimates using Climate Hazards Group InfraRed Precipitation with Station (CHIRPS) 0.05° resolution daily data. (A) As an example, CHIRPS data for the study area are shown for January 20, 2013 and (B) values within a 0.25° radius from the participant centroid are identified; (C) the mean value within the buffer for each day over the study period is shown. This process was completed for each participant using her home GPS coordinates to calculate rainfall for each day of her pregnancy.
(PNG)

S1 Table Predictors of missing antenatal care (ANC) attendance, VHEMBE study, South Africa, 2012–2013. Data are presented as mean (SD) or geometric mean (×/geometric SD) for continuous measures, and n (%) for categorical measures.
(DOCX)

S2 Table Unadjusted, complete-case comparison of truncated Fourier series models for seasonality of antenatal care attendance, maternal dietary intake, and infant birth size, VHEMBE study, South Africa, 2012–2013. Data are likelihood ratio test statistics comparing generalized linear models regressed on Fourier terms for date of birth. Four models were compared for each outcome: (1) intercept only (Null model); (2) including the first order Fourier pair (unimodal); (3) including the first and second order Fourier pairs (bimodal); and (4) including the first through third order Fourier pairs (trimodal). Likelihood ratio tests were used to compare nested models.
(DOCX)

S3 Table Relationship between daily rainfall during each trimester of pregnancy and antenatal care (ANC) attendance, VHEMBE study, South Africa, 2012–2013. Data are odds ratios (OR) and 95% confidence intervals (CI) from logistic regression models of binary antenatal care (ANC) attendance outcomes regressed on average daily rainfall (continuous mm/day) for each trimester. Inverse probability of censoring weights were applied to all models to account for missing outcomes data. Adjusted models include maternal parity, HIV status, education, marital status, and pregnancy desire; father’s supportiveness of the pregnancy; and household income and distance to a main road.
(DOCX)
