# Supplementary figures and images for: Seasonality of antenatal care attendance, maternal dietary intake, and fetal growth in the VHEMBE birth cohort, South Africa
Source: PLoS One. 2019 Sep 25;14(9):e0222888. doi: 10.1371/journal.pone.0222888 (PMC6760765; doi:10.1371/journal.pone.0222888)

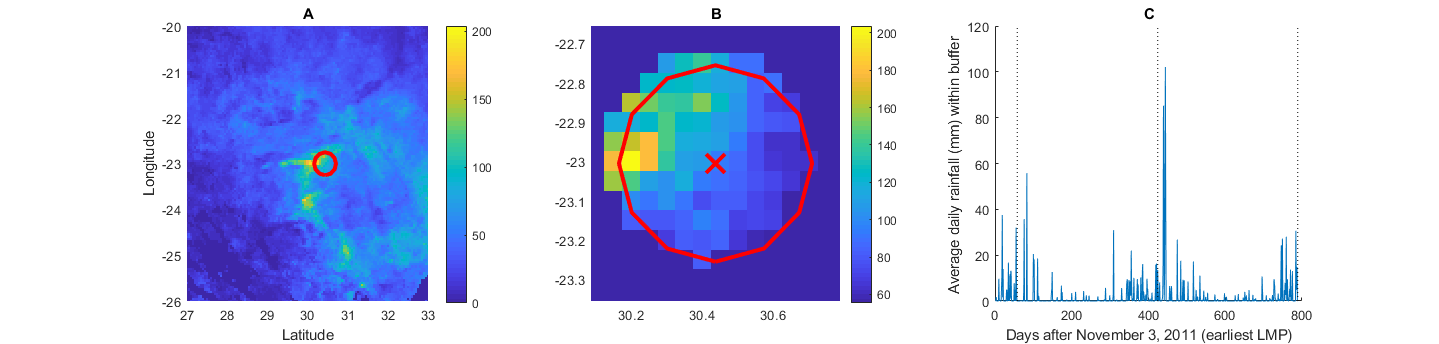

Supplement: S1 Fig — (A) As an example, CHIRPS data for the study area are shown for January 20, 2013 and (B) values within a 0.25° radius from the participant centroid are identified; (C) the mean value within the buffer for each day over the study period is shown. This process was completed for each participant using her home GPS coordinates to calculate rainfall for each day of her pregnancy. (PNG) [file pone.0222888.s001.png]
