# Supplementary material for: Seasonality of antenatal care attendance, maternal dietary intake, and fetal growth in the VHEMBE birth cohort, South Africa
Source: PLoS One. 2019 Sep 25;14(9):e0222888. doi: 10.1371/journal.pone.0222888 (PMC6760765; doi:10.1371/journal.pone.0222888)
Supplement: S1 Table — Data are presented as mean (SD) or geometric mean (×/geometric SD) for continuous measures, and n (%) for categorical measures. (DOCX) [file pone.0222888.s002.docx]

**S1 Table.** Predictors of missing antenatal care (ANC) attendance, VHEMBE study, South Africa, 2012-2013 (N=752).

|  | Antenatal care records | |  |  |
| --- | --- | --- | --- | --- |
|  | Missing | Observed | p-value | Test |
|  | N=140 | N=612 |  |  |
| Season of birth |  |  | <0.01 | Pearson's χ² |
| Harvest | 34 (24.3) | 305 (49.8) |  |  |
| Gardening | 37 (26.4) | 137 (22.4) |  |  |
| Lean/Rainy | 69 (49.3) | 170 (27.8) |  |  |
| Gestational age (weeks) | 38.96 (2.3) | 39.34 (2.3) | 0.08 | Two sample t test |
| Maternal age |  |  | 0.08 | Pearson's χ² |
| 18-21 years | 53 (37.9) | 180 (29.4) |  |  |
| 22-29 years | 58 (41.4) | 258 (42.2) |  |  |
| ≥ 30 years | 29 (20.7) | 174 (28.4) |  |  |
| Parity |  |  | 0.24 | Pearson's χ² |
| First child | 69 (49.3) | 257 (42.0) |  |  |
| Second child | 31 (22.1) | 170 (27.8) |  |  |
| ≥ Third child | 40 (28.6) | 185 (30.2) |  |  |
| Adverse prior pregnancy outcome | 14 (10.0) | 93 (15.2) | 0.11 | Pearson's χ² |
| Maternal HIV status |  |  | 0.59 | Pearson's χ² |
| HIV Negative | 116 (84.7) | 529 (86.4) |  |  |
| HIV Positive | 21 (15.3) | 83 (13.6) |  |  |
| Maternal education |  |  | 0.77 | Pearson's χ² |
| < Secondary | 77 (55.0) | 335 (54.8) |  |  |
| Secondary | 45 (32.1) | 184 (30.1) |  |  |
| Further studies | 18 (12.9) | 92 (15.1) |  |  |
| Marital status |  |  | 0.36 | Pearson's χ² |
| Not married | 78 (55.7) | 314 (51.4) |  |  |
| Married or living as married | 62 (44.3) | 297 (48.6) |  |  |
| Mother's pregnancy desire |  |  | 0.08 | Pearson's χ² |
| Wanted a baby now | 47 (33.6) | 255 (41.7) |  |  |
| Untimed or unintended | 93 (66.4) | 356 (58.3) |  |  |
| Father's support during pregnancy |  |  | 0.73 | Pearson's χ² |
| Very supportive | 106 (75.7) | 454 (74.3) |  |  |
| Somewhat or less | 34 (24.3) | 157 (25.7) |  |  |
| Religiosity |  |  | 0.06 | Pearson's χ² |
| ≥ Weekly worship attendance | 96 (69.1) | 468 (76.7) |  |  |
| ≤ Monthly worship attendance | 43 (30.9) | 142 (23.3) |  |  |
| Household monthly income (Rand) | 2059 (×/2.4) | 2163 (×/2.3) | 0.54 | Two sample t test, logged data |
| Distance from home to main road (km) | 2.70 (2.8) | 2.28 (2.6) | 0.10 | Two sample t test |

Data are presented as mean (SD) or geometric mean (×/geometric SD) for continuous measures, and n (%) for categorical measures.
