# Supplementary material for: Seasonality of antenatal care attendance, maternal dietary intake, and fetal growth in the VHEMBE birth cohort, South Africa
Source: PLoS One. 2019 Sep 25;14(9):e0222888. doi: 10.1371/journal.pone.0222888 (PMC6760765; doi:10.1371/journal.pone.0222888)
Supplement: S2 Table — Data are likelihood ratio test statistics comparing generalized linear models regressed on Fourier terms for date of birth. Four models were compared for each outcome: (1) intercept only (Null model); (2) including the first order Fourier pair (unimodal); (3) including the first and second order Fourier pairs (bimodal); and (4) including the first through third order Fourier pairs (trimodal). Likelihood ratio tests were used to compare nested models. (DOCX) [file pone.0222888.s003.docx]

**S2 Table.** Unadjusted, complete-case comparison of truncated Fourier series models for seasonality of antenatal care attendance, maternal dietary intake, and infant birth size, VHEMBE study, South Africa, 2012-2013.

|  |  |  |  |  |  |  |  |  |  |  |  |  |  |  |  |  |  |  |
| --- | --- | --- | --- | --- | --- | --- | --- | --- | --- | --- | --- | --- | --- | --- | --- | --- | --- | --- |
|  |  | 1st order (unimodal) | | |  | 1st-2nd order (bimodal) | | | | | |  | 1st-3rd order (trimodal) | | | | | |
|  |  | vs. Null: | | |  | vs. Null: | | | vs.1st order: | | |  | vs. Null: | | | vs. 1st-2nd order: | | |
| Outcome | N | LR χ² | (df) | p-value |  | LR χ² | (df) | p-value | LR χ² | (df) | p-value |  | LR χ² | (df) | p-value | LR χ² | (df) | p-value |
| Antenatal care attendance |  |  |  |  |  |  |  |  |  |  |  |  |  |  |  |  |  |  |
| ≥ 4 total visits | 612 | 3.59 | (2) | 0.17 |  | 7.80 | (4) | 0.10 | 4.21 | (2) | 0.12 |  | 10.83 | (6) | 0.09 | 3.02 | (2) | 0.22 |
| First visit ≤ 12 weeks | 605 | 2.08 | (2) | 0.35 |  | 3.39 | (4) | 0.50 | 1.30 | (2) | 0.52 |  | 4.70 | (6) | 0.58 | 1.32 | (2) | 0.52 |
| Maternal diet (% of energy) |  |  |  |  |  |  |  |  |  |  |  |  |  |  |  |  |  |  |
| Carbohydrate | 751 | 59.5 | (2) | <0.01 |  | 65.1 | (4) | <0.01 | 5.64 | (2) | 0.06 |  | 69.4 | (6) | <0.01 | 4.28 | (2) | 0.12 |
| Fat | 751 | 61.5 | (2) | <0.01 |  | 67.9 | (4) | <0.01 | 6.37 | (2) | 0.04 |  | 72.7 | (6) | <0.01 | 4.86 | (2) | 0.09 |
| Protein | 751 | 2.47 | (2) | 0.29 |  | 3.89 | (4) | 0.42 | 1.43 | (2) | 0.49 |  | 8.40 | (6) | 0.21 | 4.50 | (2) | 0.11 |
| Infant birth size (z-score) |  |  |  |  |  |  |  |  |  |  |  |  |  |  |  |  |  |  |
| Birth weight | 751 | 0.28 | (2) | 0.87 |  | 8.21 | (4) | 0.08 | 7.93 | (2) | 0.02 |  | 8.94 | (6) | 0.18 | 0.73 | (2) | 0.69 |
| Birth length | 746 | 9.15 | (2) | 0.01 |  | 18.84 | (4) | <0.01 | 9.70 | (2) | 0.01 |  | 20.94 | (6) | <0.01 | 2.09 | (2) | 0.35 |
| Head circumference | 746 | 2.26 | (2) | 0.32 |  | 10.17 | (4) | 0.04 | 7.92 | (2) | 0.02 |  | 11.90 | (6) | 0.06 | 1.72 | (2) | 0.42 |

Data are likelihood ratio test statistics comparing generalized linear models regressed on Fourier terms for date of birth. Four models were compared for each outcome: (1) intercept only (Null model); (2) including the first order Fourier pair (unimodal); (3) including the first and second order Fourier pairs (bimodal); and (4) including the first through third order Fourier pairs (trimodal). Likelihood ratio tests were used to compare nested models.
