# Supplementary material for: Seasonality of antenatal care attendance, maternal dietary intake, and fetal growth in the VHEMBE birth cohort, South Africa
Source: PLoS One. 2019 Sep 25;14(9):e0222888. doi: 10.1371/journal.pone.0222888 (PMC6760765; doi:10.1371/journal.pone.0222888)
Supplement: S3 Table — Data are odds ratios (OR) and 95% confidence intervals (CI) from logistic regression models of binary antenatal care (ANC) attendance outcomes regressed on average daily rainfall (continuous mm/day) for each trimester. Inverse probability of censoring weights were applied to all models to account for missing outcomes data. Adjusted models include maternal parity, HIV status, education, marital status, and pregnancy desire; father’s supportiveness of the pregnancy; and household income and distance to a main road. (DOCX) [file pone.0222888.s004.docx]

**S3 Table.** Relationship between daily rainfall during each trimester of pregnancy and antenatal care (ANC) attendance, VHEMBE study, South Africa, 2012-2013.

|  |  |  |  |  |  |  |  |  |  |  |  |  |  |  |  |  |  |  |  |  |  |
| --- | --- | --- | --- | --- | --- | --- | --- | --- | --- | --- | --- | --- | --- | --- | --- | --- | --- | --- | --- | --- | --- |
|  |  | 1st trimester rainfall | | | | | |  | 2nd trimester rainfall | | | | | |  | 3rd trimester rainfall | | | | | |
|  |  | Unadjusted | |  | Adjusted | |  |  | Unadjusted | |  | Adjusted | |  |  | Unadjusted | |  | Adjusted | |  |
| ANC attendance | N | OR | (95% CI) | | OR | (95% CI) | |  | OR | (95% CI) | | OR | (95% CI) | |  | OR | (95% CI) | | OR | (95% CI) | |
| ≥ 4 total visits | 612 | 1.08 | (0.91 to | 1.27) | 1.08 | (0.91 to | 1.29) |  | 1.03 | (0.95 to | 1.12) | 1.02 | (0.94 to | 1.11) |  | 0.90 | (0.83 to | 0.97) | 0.89 | (0.82 to | 0.96) |
| First visit ≤ 12 weeks | 605 | 0.92 | (0.77 to | 1.11) | 0.90 | (0.75 to | 1.09) |  | – |  |  | – |  |  |  | – |  |  | – |  |  |

Data are odds ratios (OR) and 95% confidence intervals (CI) from logistic regression models of binary antenatal care (ANC) attendance outcomes regressed on average daily rainfall (continuous mm/day) for each trimester. Inverse probability of censoring weights were applied to all models to account for missing outcomes data. Adjusted models include maternal parity, HIV status, education, marital status, and pregnancy desire; father’s supportiveness of the pregnancy; and household income and distance to a main road.
